# WC-High Entropy Alloy Reinforced Long Life Self-Grinding Silage Knife Prepared by Laser Cladding

**DOI:** 10.3390/nano12061013

**Published:** 2022-03-19

**Authors:** Lingfeng Xu, Mingxiang Li, Zhanhua Song, Fade Li, Jing Guo, Ming Gao

**Affiliations:** 1 Mechanical and Electronic Engineering College, Shandong Agricultural University, Taian 271018, China; lingfengxu@126.com (L.X.); limx1018@163.com (M.L.); lifade@sdau.edu.cn (F.L.); gjcp@sdau.edu.cn (J.G.); 2 Shandong Provincial Key Laboratory of Horticultural Machineries and Equipments, Shandong Agricultural University, Taian 271018, China; 3 Shandong Agricultural Equipment Intelligent Engineering Laboratory, Shandong Agricultural University, Taian 271018, China

**Keywords:** high entropy alloy, WC particles, self-grinding, microstructure, wear resistance

## Abstract

The working environment of agricultural knives is bad, which makes the knives wear out easily. A wear resistant layer of AlCoCrFeNi high entropy alloy (HEA) reinforced by tungsten carbide (WC) was prepared by laser cladding on one side of the cutting edge of a 65 Mn silage knife. Both the effects of WC addition on the microstructure and mechanical properties of AlCoCrFeNi (WC)_x_ (x = 0, 0.1, 0.2 and 0.3 in mass percentage) alloys were investigated. All experimental alloys displayed a crystalline structure of simple body centered cubic (BCC). The hardness of the cladding layer increases with the increase of WC content, and the hardness value enhances from 740 HV0.2 to 1060 HV0.2. A self-grinding edge was formed during working for the cladded knives. The cutting quality can be improved and the service life of agricultural knives can be increased meanwhile. The weight loss rate of untreated knives was about 2.64 times that of the cladded knives after a 76 h field experiment.

## 1. Introduction

Cutting tools are the key parts of agriculture machines, which are usually used to cut crop straws, stalks, grass, etc. in order to harvest them. The tools interact with straw, soil, etc. and are easy to wear out [1]. Dull knives lead to a reduction in cutting efficiency. In addition, the thickening cutting edge can cause crop stem splitting, fiber tissue tearing, and other cutting damage. The tool making technology of self-grinding offers a good scheme to solve the above problems [2,3]. In order to form a self-grinding edge, a layered structure which consists of a wear resistant layer and a lesser wear resistant one must be manufactured. Consequently, different amounts of wear occur at different layers of the cutting edge during working [4]. The geometry of cutting edges can remain unchanged for a long period of time, which improves the service life and cutting quality of knives [5]. Comparing the self-grinding forage harvesting knives prepared by laser cladding with ordinary knives, the wear speed of the self-grinding cutting edge radius of curvature decreased by more than 50% under the same conditions [6].

High entropy alloys (HEA) are usually composed of 5 or more metallic elements in which the proportion of each element is between 5% and 35% (atomic fraction) [7,8]. The high entropy effect of HEAs is beneficial for the formation of simple solid solution and inhibits the formation of complex intermetallic compounds. HEAs have excellent properties, such as high strength, high ductility, etc. which are suitable for structural applications [9,10]. Research has been carried out on the preparation of HEA layers on the substrate of common steel [11,12]. For example a layer of CrMnFeCoNi was made on the surface of Q235 steel by laser cladding [13]. Also, on the surface of Q235 steel a layer of CoCr_2_FeNiMo_x_ was made to achieve high wear resistance [14]. However, the hardness of some HEAs is low and does not satisfy hardness and wear resistance requirements of agriculture knives [15]. So, the addition of hard ceramic particles becomes an effective method to increase hardness and wear resistance of HEAs [16,17].

The ceramic particle of WC is one of the most used hard phases due to its excellent properties [18,19]. WC is tungsten carbide which is a compound composed of tungsten and carbon with structure of hexagonal crystal. The hardness of WC is between 16 GPa and 22 GPa [20]. The wettability of WC with metal matrix is good. The hardening effect of WC on matrix is remarkable. On the other hand, the nailing effect produced by hard particles can limit grain growth and promote the hardness of metal matrix [21]. A coating of HEA reinforced with WC was prepared by plasma cladding to increase wear resistance [22]. The effect of WC content on microstructure and mechanical properties of composite coatings was analyzed. The results show that the existence of WC particles can effectively alleviate the wear damage of the coating and then significantly improve the wear-resistant properties of the coating. Although the addition of ceramic particles leads to an improvement of HEAs’ mechanical properties [23,24], the action mechanism of ceramic particles on microstructure and properties of HEAs is still not completely clear. In addition, few studies have been carried out on the application of ceramic particle reinforced HEAs in agricultural knives.

Laser cladding, as an advanced method of coating preparation, can achieve metallurgical bonding between the coating material and substrate, which is important for the preparation of agricultural knives in harsh working conditions [25]. It has the characteristics of low dilution rate, high bonding strength, and fast solidification [26]. The short process time of preparing HEA by laser cladding can offset the shortcomings of the traditional methods. So, laser cladding was used in this study to prepare the HEA layer on silage knives. AlCoCrFeNi is a representative HEA system, which is considered as a kind of coating material that can be deposited on traditional alloys to improve their hardness and wear resistance [27]. The WC particles were added in the AlCoCrFeNi HEA, and then were coated on one side of the cutting edge of 65 Mn steel by laser cladding. The effect of WC on performance of the AlCoCrFeNi HEA coating was studied, and its microstructure, hardness and wear resistance were carefully analyzed in order to develop an alloy coating with high wear resistance. Finally, the knives cladded with WC-AlCoCrFeNi composites were tested in the field. In this work, the strengthening mechanism of WC on HEA was discussed and the application range of WC reinforced HEA was expanded. This work also provides new material and technology for the preparation of self-grinding cutting tools.

## 2. Materials and Methods

### 2.1. Experimental Materials

**Figure 1:**
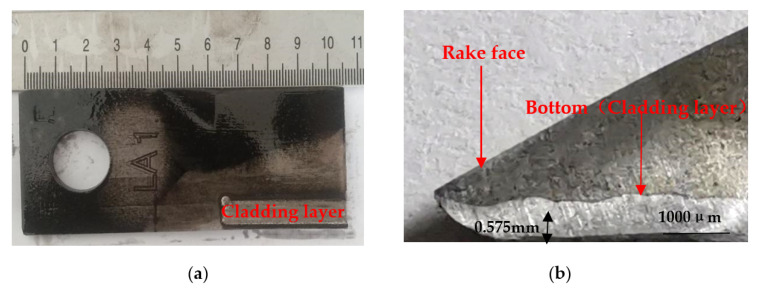
Knife: (**a**) macro-morphology of the silage knife after cladding; (**b**) cross-section morphology of the knife’s edge after cladding.

Figure 1 shows the experimental knife of 65 Mn steel used in lawn mower, whose components are shown in Table 1. Figure 1a shows the underside of the whole knife after cladding, and Figure 1b shows the section of the cutting edge with cladding layer. The HEA powder of AlCoCrFeNi with WC was cladded at the bottom surface of cutting edge to form hardness gradient. The weight loss on the bottom surface was smaller than the rake face during working. The cladded wear resistant material protruded at the front edge to form a self-grinding edge. Before laser cladding, the knives surface was polished with sandpaper to remove the oxidation layer and washed with 99.5% acetone solution. The cladding powder consisted of Al, Co, Cr, Fe and Ni powder with the equal mole ratio. WC powder was added to AlCoCrFeNi powder with a mass fraction of 0, 10%, 20% and 30%, respectively. The alloy powder was mixed in a powder mixer of V type for 27 h.

### 2.2. Laser Cladding

Laser cladding was carried out in the 3D rapid molding remanufacturing system, YLS-4000 (IPG Photonics Corporation, Beijing, China). The cladding alloy powder was dried at 100 °C for 30 min in drying oven to remove moisture in the alloy powder and to increase its liquidity. Argon was used as protective gas to isolate oxygen in the process of cladding and as the means to transport cladding powder to the surface of substrate. The speed of the powder delivery device was 1.3 r/min. The spot diameter of the laser was 3 mm with a scanning speed of 0.03 m/s. The overlap ratio of the cladding was 50%, and the laser power was 1600 W. Keeping above cladding parameters, the cladding alloy powders with different contents of WC were cladded and analyzed.

### 2.3. Microstructure Analysis

After cladding, samples were cut from knives perpendicular to the laser scanning at an wire cutting machine (DK7720, Kunshan, Jiangsu, China). Allowable deviation of this wire cutting machine is plus or minus 0.005 mm. After mounting, grinding and polishing, the samples were corroded with aqua regia for 15–20 s. Then, they were washed with absolute alcohol. The microstructures of cladding layers and the interface between the cladding layer and substrate were observed by a metallographic microscope (CAI Kang 4XCE, CAI Kang Optical Instrument Co. Ltd., Shanghai, China) and a field e mission scanning electron microscope (ZEISS UTRAL55, Rotterdam, Netherlands). The chemical composition of the micro-zone of cladding layers was analyzed with an energy-dispersive spectrometer. The phase of cladding layers was determined using an Empyrean X-ray diffractometer (ZEISS UTRAL55, Rotterdam, Netherlands) which had a scanning velocity of, °/min from 20° to 110°.

### 2.4. Hardness and Wear Resistance Analysis

The hardness of cladding layers was measured using a digital micro hardness tester (TMVP-1, Beijing Times Peak Technology Co. Ltd., Beijing, China), whose accuracy is plus or minus 4%. The hardness test positions were carried out along the depth direction of the cladding layer. The distance between adjacent test points was 100 μm, and the test range was from the cladding layer to the substrate.

The friction and wear tests of the samples were carried out by a friction and wear testing machine (MMS-2A, Jinan Yihua Tribology Testing Technology Co. Ltd., Jinan, China) at room temperature. The maximum test force is 2000 N, the allowable deviation of test force is plus or minus 2% and the allowable deviation of friction torque is plus or minus 3%.

The samples were prepared according to the GB/T 3960–2016 standard. The sample size was 30 mm × 7 mm × 6 mm. The grinding surface of test was 30 mm × 7 mm along the cladding layer. The test load exerted on the samples was 100 N, and the rotation speed was 200 r/min. The counterpart of tested samples was a steel with 0.45% carbon, whose diameter was 40 mm. The mass loss of samples was measured once an hour, and the wear time for each sample was 5 h. The mass loss was weighed by the electronic balance, whose accuracy is 0.0001 g.

### 2.5. Field Test

The field test site was Maodeng Pasture at Inner Mongolia Autonomous Region (Xilin Gol League, Inner Mongolia, China). The experimental harvest object was oat grass, with a height of about 120 cm and a diameter of 3–4 mm. The oat grass harvested in the experiment was mainly used as tempered hay. The test equipment is a lawn mower of GMT-3605FL produced by JF-Stoll Company in Copenhagen Denmark. There are 9 cutter heads with 2 axes for each head in one lawn mower. The working speed of lower mower was 20 Km/h, the cutter head speed was 2000 r/min and the stubble height was 50–150 mm. The cladded knife and the untreated knife were installed on the same cutter head for field mowing test under the same conditions. The wear weight loss and cutting edge morphology of knives was measured and compared.

## 3. Experimental Results and Discussion

### 3.1. Optical Morphology

**Figure 2:**
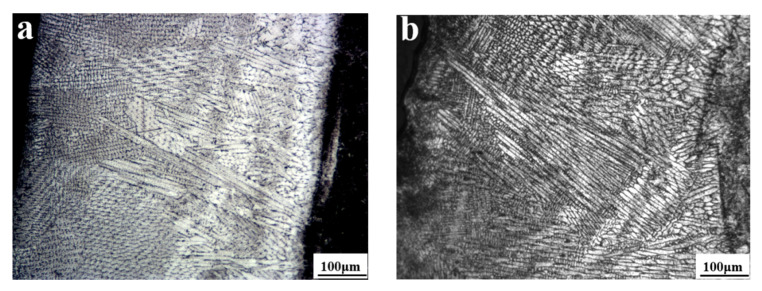

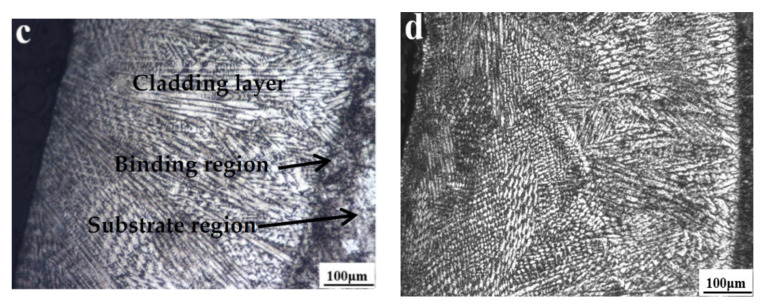
Metallographic structure of cladding layers: (**a**) microstructure of the cladding layer of AlCoCrFeNi(WC)_0_ and substrate; (**b**) microstructure of the cladding layer of AlCoCrFeNi(WC)_0.1_ and substrate; (**c**) microstructure of the cladding layer of AlCoCrFeNi(WC)_0.2_ and substrate; (**d**) microstructure of the cladding layer of AlCoCrFeNi(WC)_0.3_ and substrate.

As a hard phase, WC is usually added to the alloy to improve its hardness and wear resistance. The AlCoCrFeNi(WC)_x_ (x = 0%, 10%, 20% and 30% WC) were coated on the bottom face of 65 Mn cutting edge at the cladding power of 1600 W. Figure 2 shows the section morphology of AlCoCrFeNi(WC)_x_, respectively. Under the selected process parameters, the crystal of cladding layers of AlCoCrFeNi(WC)_x_ possesses obvious directionality, which is a typical quick directional solidification microstructure. The cladding layers are mainly composed of columnar crystal, equiaxed crystal, and cellular crystal [28]. At the bottom of cladding layers, the microstructure was mainly composed of directionally solidified columnar crystals. The secondary columnar crystal was developed at the middle part of cladding layers, and the dense equiaxed crystal structure was present at the top of the cladding layer. It can be seen that metallurgical bonding between the cladding layer and substrate was formed during cladding. The interface between the cladding layer and the substrate was kept straight and clear, wherein flat crystals were formed.

### 3.2. X-ray Diffraction Analysis

**Figure 3:**
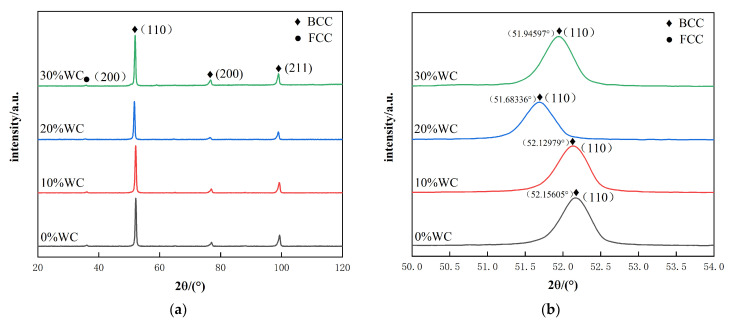
XRD image: (**a**) XRD patterns of AlCoCrFeNi(WC)_x_ alloys; (**b**) the detailed scans for the peak of (110) of BCC solid solution phase.

The X-ray diffraction patterns of AlCoCrFeNi alloys with different WC contents are shown in Figure 3a. All alloys exhibit the reflections of a structure, which is mainly consisted of a body centered cubic solid solution with a small amount of face centered cubic solid solution. It can be seen that the addition of WC has no significant effect on the crystalline structure of AlCoCrFeNi alloy. Figure 3b shows the detailed scans for the highest peak of (110) of BCC solid solution phase. The peak of (110) shifts to the lower 2θ with the addition of WC. This change is identical to the variation of the lattice constants of the BCC phase, which are calculated with the strongest peak of (100). The values of the lattice constant 0.28724, 0.28753, 0.28841, and 0.28787 nm in the AlCoCrFeNi(WC)_x_ alloys with x = 0, 0.1, 0.2, and 0.3, respectively. It was found that the lattice constants increased with the addition of WC content. WC was decomposed during the cladding process and W/C element was dissolved in the BCC lattice [29]. The atomic radius of the experiment elements is summarized in Table 2. The W element has the second largest atomic radius in the seven elements, which would lead to the lattice distortion that corresponds to the increased lattice constant [30]. At 20%, the mutation of FCC lattice constant is obvious and the lattice distortion is significant.

### 3.3. SEM and EDS Analysis

**Figure 4:**
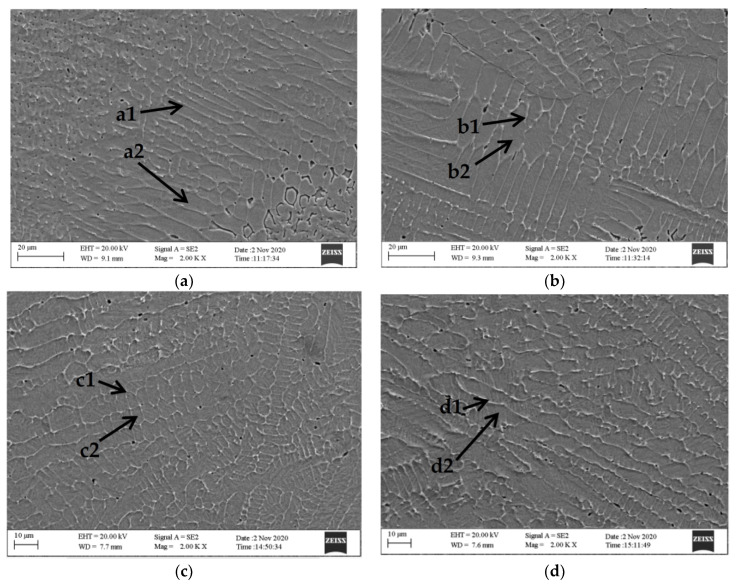
SEM images of cladding layers: (**a**) the cladding layer of AlCoCrFeNi(WC)_0_; (**b**) the cladding layer of AlCoCrFeNi(WC)_0.1_; (**c**) the cladding layer of AlCoCrFeNi(WC)_0.2_; and (**d**) the cladding layer of AlCoCrFeNi(WC)_0.3_.

Figure 4 is the SEM diagrams of the middle part of the cladding layers corresponding to AlCoCrFeNi alloys with different WC contents. The difference of WC content led to the difference of the microstructure and morphology of the cladding layer. Laser cladding was a non-equilibrium solidification process of rapid heating and cooling. At the same time, WC decomposed at high temperature and caused the increase of nucleation in the cladding layer, affecting the growth direction of the internal crystals. As shown in the figure, with the change of WC content, grains of the cladding layer were finer with high WC content than that with low WC content, and the intergranular structure was greater. With the increase of WC content, the hardness and wear resistance of the cladding layer increased.

Table 3 shows the EDS component analysis results of the cladding layers of AlCoCrFeNi(WC)_x_. There are different degrees of segregation in all cladding layers. The addition of WC increased the distribution difference of alloy elements between crystals and inner crystals. WC was decomposed during the cladding process, and part of W/C element was dissolved inside grains, leading to lattice distortion (Figure 3b). However, when WC content was 30%, the solution amount of W element in the grain did not increase with the increase of WC content. Table 4 shows the enthalpy of mixing between alloy elements [31]. The enthalpy of mixing between element C and other elements is negative, especially between C and W and Cr and Fe. Thus, C easily forms a stable phase with W, Cr, and Fe. The contents of W, Cr, and Fe elements in crystals are lower than those between crystals, and the hard phases of C, W, Cr, and Fe are formed, which improves the hardness of cladding layer [32].

### 3.4. Hardness Analysis

**Figure 5:**
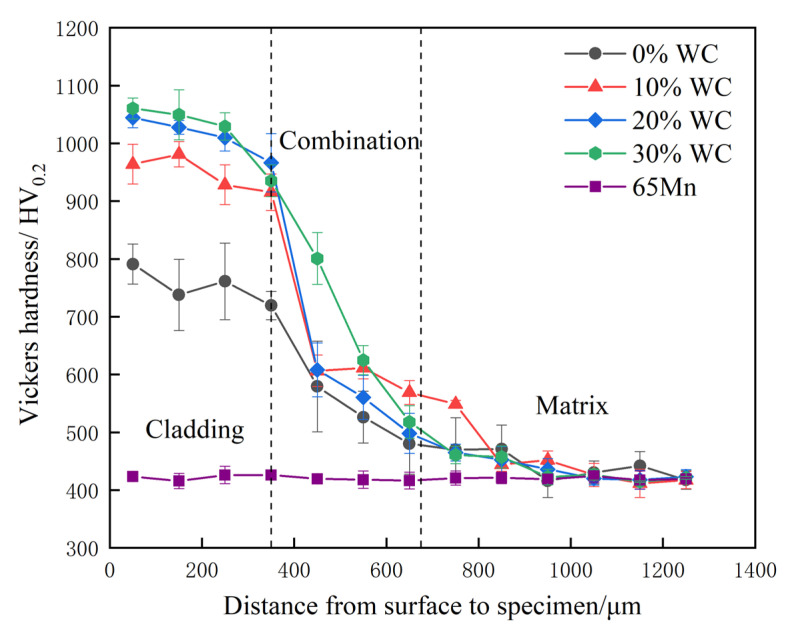
Effect of WC content on the microhardness of cladding coating.

The hardness distribution curve of all cladding layers is shown in Figure 5. The cladding layer hardness of AlCoCrFeNi HEA is between 700 HV0.2 and 800 HV0.2. After adding WC, the hardness of the cladding layer is increased significantly. The maximum hardness is 1060 HV0.2, which is 2.52 times than that of 65 Mn substrate (420 HV0.2). A metallurgical bond was formed at the binding zone between cladding layer and substrate for all experimental alloys, and the hardness appeared as a graded distribution along the thickness. Part of cladding layer material entered into the bonding zone, which increased the hardness of the bonding zone. Especially after the addition of WC, the strengthening effect of alloys to the bonding zone was more significant. The addition of WC reduced the grain size of cladding layer and improved their metallurgical structure. A similar experimental result has been reported in Ref. [33]. During cladding, WC was decomposed under the action of laser. Part of W and C atoms dissolved into BCC lattice of AlCoCrFeNi alloy, which resulted in lattice distortion and increased the hardness of cladding layer. The undissolved W, C with Cr, and Fe tended to segregated at grain boundary and formed a hard phase, which also increased the hardness of cladding layer.

### 3.5. Wear Resistance Analysis

**Figure 6:**
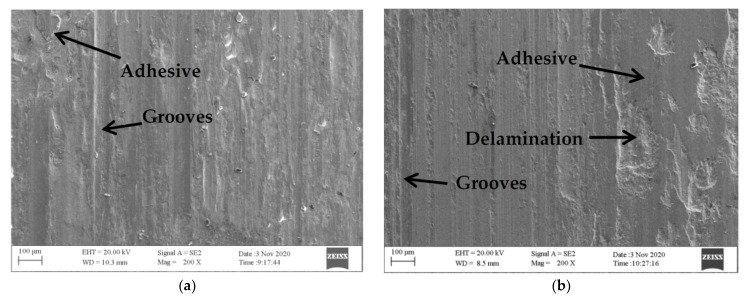

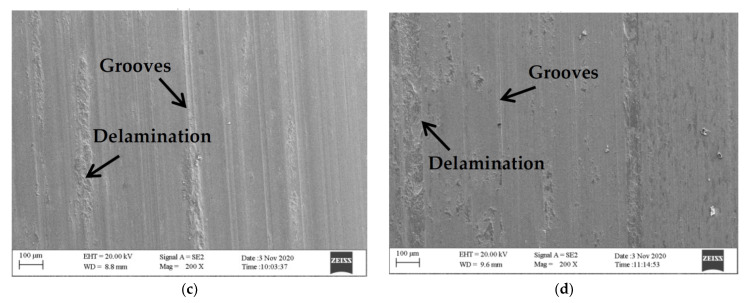
Wear morphology of AlCoCrFeNi(WC)_x_: (**a**) AlCoCrFeNi(WC)_0_; (**b**) AlCoCrFeNi(WC)_0.1_; (**c**) AlCoCrFeNi(WC)_0.2_; and (**d**) AlCoCrFeNi(WC)_0.3_.

The friction and wear tests were carried out and the worn surface morphology of the experiential alloys is shown in Figure 6. Friction test exerted a serious plough effect on surface of cladding layers. Deep grooves and delamination were made on surface of HEAs with 0% and 10% WC, the worn surface was rough, and there was a large area of adhesive layer. Under the friction stress the adhesive layer eventually tore and peeled off, which resulted in pits on the surface. It can be seen that the wear was severe on the surface of the alloys with low content of WC, and wide adhesive worn areas and delamination were observed. With the increase in the content of WC, the resistance of cladding layer to plastic deformation strengthened and the adhesion of friction surface attenuated. The grooves became shallow and the area of severe delamination shrank. The groove became the main wear form, which showed the occurrence of abrasive wear [28].

**Figure 7:**
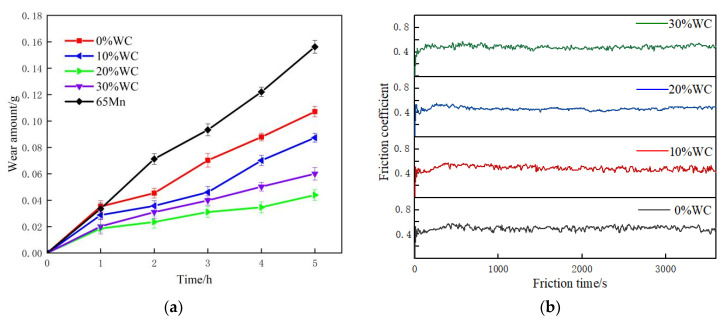
Effect of WC content on wear properties of cladding layer: (**a**) the weight loss of cladding layer; (**b**) the friction coefficient curve of cladding layer.

Figure 7a shows the relation curve between wear weight loss and friction time of the AlCoCrFeNi(WC)_x_. The weight loss of four cladding layers was 0.105 g, 0.086 g, 0.043 g, and 0.060 g, respectively, which decreased first and then increased with the increase of WC content, corresponding to the friction coefficient curve (Figure 7b and Table 5). The weight loss of AlCoCrFeNi(WC)_0.2_ was the minimum. As can be seen from the Figure 6, the hardness of cladding layers increased with the increase of WC content. The relationship between the hardness and the wear loss did not conform to the Archard rule completely, in which the wear loss decreased with the increase of the material hardness. The Archard rule is mainly based on single wear mechanism, such as adhesion wear or abrasive wear [34]. In this work, the wear mechanism was composed of adhesive wear and abrasive wear [35]. The mechanical properties and microstructure of the material affected its wear mode, which changed with the addition of WC.

In fact, the wear resistance of materials does not depend on hardness completely, and other properties such as toughness can also apply effects on it [36]. On the one hand, an appropriate amount addition of WC can refine the microstructure and improve the hardness of cladding layer, which improved wear resistance of material. The friction coefficient of the cladding layer decreased, the friction coefficient curve was smoother, and the vibration amplitude was reduced. On the other hand, the addition of WC reduced the toughness of material, which resulted in the decrease of wear resistance. So, the hardness and toughness of cladding layer can all be taken into account by adding 20% WC, which was also consistent with our previous test results [37].

## 4. Field Test

**Figure 8:**
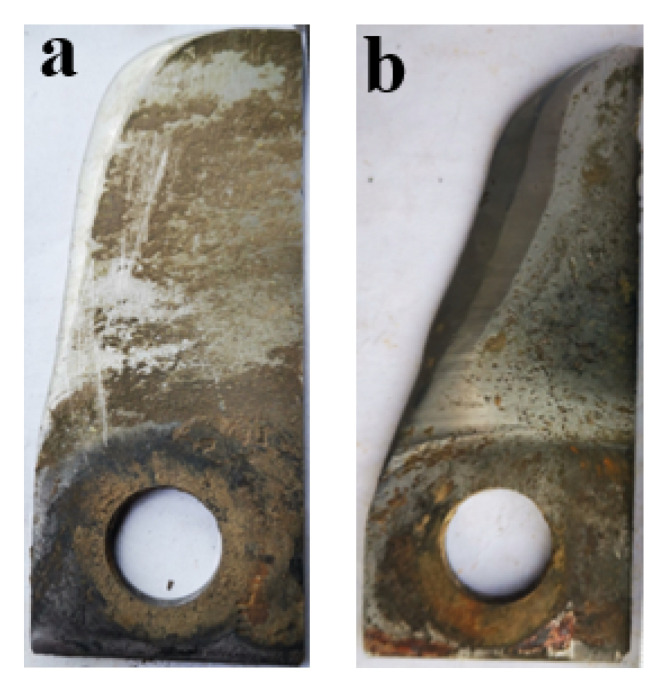
Macro morphologies of the two knives after 50 h of operation: (**a**) knife with laser cladding; (**b**) knife without laser cladding.

The macromorphology of the self-grinding knife cladded with AlCoCrFeNi(WC)_0.2_ and the knife of 65 Mn steel without cladding were shown in Figure 8 after 50 h operation. The cutting direction was indicated by the arrow in Figure 8. The knife point (upper left corner of the shown knife) of the cladded knife was worn, but the rectangular shape remains. The knife without laser cladding was severely worn and has lost its original shape. The cutting edge of the tool was passivated. High working speed of rotary flail knives leads to severe wear in field operation. In particular, the knife point with the fastest cutting speed and the longest cutting distance is more likely to wear than other parts of the knife due to the longest distance between the knife point and the rotary shaft, which leads to the reduction of cutting performance. The self-grinding knife cladded with AlCoCrFeNi(WC)_0.2_ and the knife without cladding were installed on one rotating shaft, so they worked at the same working conditions. For the self-grinding knife, the wear quantity of two surfaces of one cutting edge was different. The cladding layer protruded from the blade of knife and self-grinding edge formed. Compared with the self-grinding knife, the wear resistance of two surfaces of one cutting edge of knife without cladding was low and the same. The knife point away from the knife shaft wore out easier and was unable to maintain the original shape. The cutting performance became poor and the cutting damage to the stem became serious [38].

**Figure 9:**
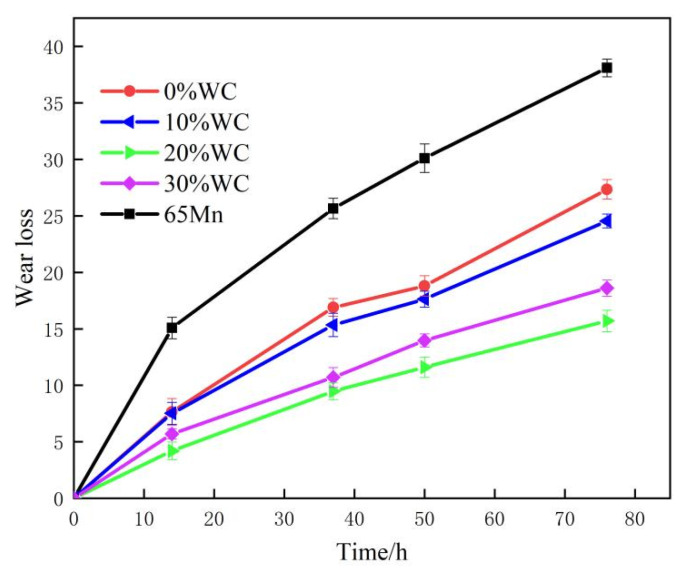
Comparison of wear loss in field tests.

All test knives were assembled on the same equipment. Under the same working conditions, the weight loss of each knife along with working time was recorded, as shown in Figure 9.

The wear rate of knives was high at the initial operation stage. As the operation time increases, the wear of knives became normal and the wear weight loss rate decreased. The weight loss of cladded knives was obviously lower than that of the knife without cladding. After 76 h of operation, the weight loss rate of the knife cladded with AlCoCrFeNi(WC)_0.2_ is only 0.192 g/h, while the weight loss rate of the knife without cladding was 0.506 g/h. The cladding layer with compact structure was firmly combined with the substrate, and the cladding layers of all cladded knives did not fall off during the working process. Different WC content in cladding layers leaded to different weight loss of knives, and the weight loss of knife cladded with AlCoCrFeNi(WC)_0.2_ was the lowest.

Laser cladding technology can not only meet the requirements of HEA preparation reinforced by WC, but also accurately control the thickness of the cladding layer to meet the requirements of self-grinding knives. In terms of economic benefits, laser cladding increased the manufacturing cost of agriculture knives by about thirty percent. However, the working life of cladded knives was extended above 2.5 times. In addition, crop cutting quality was improved and decreased auxiliary time of the crop harvest.

## 5. Conclusions

The layers of AlCoCrFeNi(WC)_x_ (x = 0, 0.1, 0.2 and 0.3 in mass percentage) were made on 65 Mn steel by laser cladding. The addition of WC did not change the lattice type of AlCoCrFeNi. A typical fast directional solidification structure with uniform composition was formed in the cladding layers of all of the alloys.

(1) In the process of laser cladding, WC particles decomposed and part of W/C atoms dissolved into the lattice of HEA, which resulted in lattice distortion. The addition of WC refined the microstructure of cladding layer and improved its hardness. The hardness was up to 1060 (HV0.2) for the cladding layer;
(2) Although the addition of WC increased wear resistance of AlCoCrFeNi alloy, the wear loss did not decrease with the increase of WC content. The wear resistance of AlCoCrFeNi(WC)_0.2_ was the best.
(3) The self-grinding edge can formed during operation for knives cladded with the AlCoCrFeNi(WC)_x_. The life of the knives was significantly improved.

## Figures and Tables

**Table 1:** 65 Mn chemical composition of the matrix material.

Composition	C	Si	Mn	S	P	Cr	Ni	Cu
Content (wt%)	0.64	0.23	1.15	0.028	0.032	0.23	0.15	0.2

**Table 2:** Properties of the experimental elements (from the periodic table software).

Element	Atomic Radius (pm)	Pauling Electronegativity
Al	143	1.61
Co	125	1.88
Cr	128	1.66
Fe	127	1.83
Ni	125	1.91
W	141	2.36
C	86	2.55

**Table 3:** Analysis of EDS composition in different micro areas of test alloys.

WC Content	Location	Al	Co	Cr	Fe	Ni	W	C
0	a1	6.52	13.49	13.75	44.60	13.96	0	7.47
	a2	6.01	13.88	12.61	48.64	12.63	0	6.22
10%	b1	5.04	11.56	11.05	54.17	11.67	0.35	5.61
	b2	2.81	12.02	11.24	58.17	9.99	0.26	5.02
20%	c1	2.28	3.90	5.00	75.39	3.84	0.41	9.18
	c2	2.16	3.98	3.90	80.83	3.50	0	5.36
30%	d1	2.89	5.79	15.73	58.12	5.34	2.76	9.36
	d2	3.30	6.73	6.31	70.06	6.26	0.38	6.96

**Table 4:** Mixing enthalpy between elements.

Mixing Enthalpy	Al	Co	Cr	Fe	Ni	C	W
Al	/						
Co	−19	/					
Cr	−10	−4	/				
Fe	−11	−1	−1	/			
Ni	−22	0	−7	−2	/		
C	−36	−42	−61	−50	−39	/	
W	26	−1	1	0	−3	−60	/

**Table 5:** Average friction coefficient of HEA cladding layers with different WC content.

Content of WC/%	0	10	20	30
Average friction coefficient	0.506	0.485	0.472	0.480

## Data Availability

The data presented in this study are available on request from the corresponding author.
